# The role of the university in recovering from COVID-19 and preparing for future crises—perspectives and experiences from Sunway University, Malaysia

**DOI:** 10.3389/fpubh.2023.1072823

**Published:** 2023-04-24

**Authors:** Elil Renganathan, Renzo Guinto, Jemilah Mahmood, Oliver Lacey-Hall, Abhi Veerakumarasivam, Sibrandes Poppema

**Affiliations:** ^1^School of Medical and Life Sciences, Sunway University, Bandar Sunway, Selangor, Malaysia; ^2^Sunway Centre for Planetary Health, Sunway University, Bandar Sunway, Selangor, Malaysia; ^3^Sunway University, Bandar Sunway, Selangor, Malaysia

**Keywords:** COVID-19, education, research, public health, planetary health, resilience, communications, university

## Abstract

This article is part of the Research Topic ‘Health Systems Recovery in the Context of COVID-19 and Protracted Conflict’.

Universities, as engines of knowledge creation and dissemination and as incubators of disciplined yet original thinking, have a key role to play in tackling the most complex challenges that societies and our planet face, from infectious diseases to the climate emergency. This commentary presents the perspectives from Sunway University, a young private university in Malaysia that made a strong commitment to the sustainable development goals (SDGs) prior to the pandemic, and its experiences in promoting research, innovation, and learning as part of COVID-19 recovery and in preparation for future crises such as the climate emergency. Some of the university’s initiatives include embracing the planetary health approach, reviving essential public health functions, exploring pandemic resilience, addressing ‘infodemics’ and promoting science diplomacy. The example of Sunway University provides some insights on the opportunities and challenges that academic institutions face as they seek to reorient the paradigm of education, research, and service away from disciplinary siloes and towards a more integrated, preventive, accessible and translational approach.

## 1. Introduction

The COVID-19 pandemic exposed the many gaps and challenges in the global health system, from the inability to halt the transmission of the virus and the failure to ensure access to life-saving vaccines for everyone, especially for people in low- and middle-income countries. Pre-pandemic efforts to strengthen health systems were not sufficient to achieve universal health coverage (UHC), which is an important ingredient for health system resilience in the face of shocks and stresses such as a pandemic (1). Meanwhile, lack of prioritization and weak investments in health security across countries limited their ability to adequately prepare for and respond to the outbreak. In all these global health failures, it is the most vulnerable, the poorest, and the marginalized who have faced the greatest disadvantage, and the resulting gap between the ‘haves’ and ‘have nots’ continues to grow as a result.

To worsen the situation, a lack of focus on tackling the upstream drivers of infectious disease emergence increases the likelihood of another pandemic happening within this century. Due to the slow pace of worldwide action to reduce greenhouse gas emissions, the climate emergency is already negatively impacting human health, including triggering more zoonotic spillovers thus increasing pandemic risk (2). Other planetary crises such as biodiversity loss and plastic pollution are jeopardizing human health, especially but not exclusively in poor and marginalized populations. What complicates this scenario is the emergence of mis/disinformation epidemics that negatively influence human behavior and decision-making and impede timely and urgent action.

These are the current and emerging challenges that academia, as the engine of knowledge creation and dissemination, must urgently respond to and prepare for, respectively. During this pandemic, universities have shown their adaptiveness, as demonstrated by the rapid and widespread adoption of digital technologies for teaching (3), their responsiveness to the crisis, from modeling the spread of the disease, recommendations on behavioral and social measures, and accelerated discovery and development of pandemic tools such as vaccines (4), to the provision of technical advice to governments. These achievements must be sustained and accelerated by universities as they embark on the road to post-COVID-19 recovery and prepare for future crises.

Sunway University, established in 2011, is one of Malaysia’s leading private universities. It is a strictly not-for-profit institution and dedicated to quality education, supporting enterprise, and undertaking research focused on key global problems. The University is relatively young by global standards but already ranked within the top 2 percent of universities in the world (QS World University Rankings and The Times Higher World University Ranking). It is ranked 122^nd^ in the QS Asia University Rankings 2023, as well as being ranked within the top 150 universities in the world under 50 years old.

This commentary presents perspectives from the university, which had already forged a strong commitment to the SDGs prior to the pandemic, and its experiences in promoting research, innovation, and learning as part of COVID-19 recovery and in preparation for future crises such as the climate emergency. The examples from Sunway University provide some insights on the opportunities and challenges that academic institutions face as they seek to reorient the paradigm of education, research, and service away from disciplinary siloes and towards a more integrated, preventive, accessible and translational approach.

## 2. The planetary health approach

Humanity’s continued susceptibility to infectious disease pandemics, as well as the advent of worsening ecological crises such as the climate emergency, requires a new approach that acknowledges the interconnectedness of these global problems and their underlying root causes. These challenges reveal that the health of people and the planet are inextricably intertwined; people’s health cannot be fully protected from pandemics and climate impacts if the health of the planet is ignored. In response, one approach that has emerged in recent years is planetary health, which is a scientific field, global movement, and problem-solving approach focused on understanding and addressing the growing human health impacts of anthropogenic global environmental change (5). Central to this approach is the recognition that many of the health harms that humanity is facing today are a result of human activities. Hence, at the heart of solving planetary health damage is stopping it at the source—be it rapid deforestation that disturbs pathogen-carrying wildlife or greenhouse gas emissions from humanity’s profligate use of fossil fuels.

As a biodiversity hotspot, Southeast Asia is a strategic location to advance the planetary health approach and ensure that it is integral to crisis prevention at local, regional, and global levels. In mid-2021, Sunway University established the Sunway Center for Planetary Health, aiming to pioneer the application of the planetary health approach to pressing health threats in the region (6). For its inaugural phase, the Center chose to tackle four priority themes that are highly relevant to the region: preventing the next pandemic; tackling the climate emergency; creating healthy cities; and achieving sustainable food systems. In order to address these themes, the Center also focuses on three cross-cutting enablers—governance, communications and education. The ultimate goal of planetary health is to usher the “Great Transition” of the social and economic systems that currently drive planetary health problems (7), which means that these three enablers of transformational change must not be ignored.

During its first year, the Center has closely engaged with the Malaysian government and other local, regional and international partners to help embed planetary health in policymaking and advocacy, including in supporting crafting the country’s 12th Malaysia Plan and National Planetary Health Action Plan, and piloting the Doughnut Economy model (8) in Ipoh city (Malaysia’s 4th largest city). The approach taken by Ipoh City Council emphasizes use of a regenerative economics approach to development and incorporates recognition that human health and wellbeing can and must thrive without transgressing planetary boundaries. This pilot project, the first of its kind in Asia, was launched in 2022 to support achievement of the sustainable development goals and demonstrate that living within the safe limits defined by the planetary boundaries is possible in urban environments in Asia. The Center has also deepened the discourse around reorienting the humanitarian sector towards a more anticipatory planetary health approach, and is using its advocacy and communications capacity to promote planetary health across society in Malaysia and the region.

In order to prepare the next generation, who will be on the frontlines of planetary health challenges, the Center developed a core course entitled “Community Service for Planetary Health,” which will expose all students at Sunway University to planetary health regardless of discipline. The Center has collaborated with various organizations to organize youth townhalls, bootcamps, and hackathons, which cultivate leadership and entrepreneurship for planetary health among young people.

The Sunway Center for Planetary Health is a new but active member of the Planetary Health Alliance (9) and will host the Alliance’s annual meeting in 2024 in Asia, a region not only at risk of climate and other disasters but also the epicenter of many disease outbreaks, while also serving as the global economic powerhouse of the future. The focus of the meeting will be to connect academic discourse with public participation in the planetary health movement, with the theme “From Evidence to Action: Confronting Reality.”

## 3. Essential public health functions

The COVID-19 pandemic has revealed weaknesses in social and health systems stemming from weak public health capacities in most countries. In this context, “essential public health functions” (EPHFs) are being revitalized to support an integrated approach to ensuring more sustainable and resilient health systems. In particular, World Health Organization (WHO) resolution WHA69.1 calls for strengthening essential public health functions as a basis for improving public health practice and building resilient health systems capable of meeting Universal Health Coverage (UHC) goals (10). The set of 12 EPHFs being promoted by WHO are seen as minimum requirements for countries to ensure public health and are generally regarded as a fundamental and indispensable set of collective actions under the responsibility of the state which are needed to meet public health goals, including the attainment and maintenance of the highest level of population health possible within given resources (11).

Many, if not all, postgraduate/master’s degree programs, including the most prestigious ones in the world, are based on traditional public health thinking and approaches. An extensive review of over 30 well known Master’s in Public Health (MPH) and Master’s of Global Health programs in North America, Europe and Asia-Pacific showed that none of their curricula had modules specifically dedicated to EPHFs (12). There is a clear need to establish a programme that addresses principles, theory and practice that is based on essential public health functions. The COVID-19 pandemic demonstrated that this is the public health training programme that the countries need moving forward. In response, Sunway University is developing an innovative Master’s in Public Health programme geared towards establishing competencies in support of EPHFs.

The programme will contribute to developing a workforce that can deliver the full range of EPHFs, including emergency preparedness and response, and be competent in other critical areas such as planetary health, global health and health diplomacy. It is also clear that the next generation of public health leaders needs to be equipped with practical skills necessary for navigating crises such as leadership, communications, and entrepreneurship. Thus, the planned MPH programme will draw expertise from the different schools and departments of Sunway University, including business, psychology, and communications, as well as from professionals and experts from around the world who will bring practical insights to the classroom.

This unique programme, working with international public health institutions, WHO collaborating centers, the WHO Academy and health care innovation hubs for internships, trainings and fellowships, will provide students with additional international exposure. It will produce graduates who are not only equipped with principles and theory, but with the tools and experience to operate immediately in public health practice nationally and internationally. Furthermore, the new MPH should also contribute to the Malaysian Government’s plans to future-proof healthcare, which gives more prominence to public health as means to achieve UHC and SDG 3 (Good Health and Well-being).

## 4. Resilience to future pandemics

The COVID-19 pandemic was not only a wake-up call but also an opportunity to define how humanity responds to future pandemics and the climate emergency. Thus, it is important for academic institutions to harvest the lessons from the pandemic and begin incorporating them into existing structures and systems. Sunway University is collaborating with the Universiti Kebangsaan Malaysia (UKM) to establish the Pandemic Resilience Institute. This public-private partnership harnesses the unique strengths of both institutions for mutual and greater good. By investing in the promotion of cross-institutional and multi-sector transdisciplinary collaboration, we hope to improve our biomedical understanding of disease pathogenesis and clinical outcomes as well as develop pragmatic interventions to enhance the socio-economic resilience of local communities to the random and chronic disruptions caused by pandemics.

Although significant gaps in available local health and socio-economic data have always impeded the provision of equitable, accessible, well-connected and coordinated care across all aspects of an individual’s health and social needs, the pandemic exacerbated this challenge. A key area of investment, which this collaboration focuses on, is the development of long-term studies that capture and monitor data on risk factors and health outcomes as well as the effect of specific health promotion interventions. This initiative will be approached through robust university-industry-public-environment interactions often referred to as the quadruple helix model in knowledge economy (13); especially through health promotion activities for the public through collaboration with various non-governmental organizations, particularly those working in the areas of health, climate and youth, policy recommendations and formulations with key governmental policy stakeholders and agencies, and demand-driven solution creation for key industries.

## 5. ’Infodemics‘ and science diplomacy

Finally, as alluded to earlier, clear and effective communication is a vital ingredient in crisis response, whether information is targeted to policymakers, international partners, or the general public. Unfortunately, mis/disinformation, especially but not exclusively delivered *via* social media, derailed progress in expanding vaccine coverage and eroded trust between leaders and citizens (14). In response to this challenge, Sunway University contributed to a study on ‘Addressing inaccurate and misleading information about biological threats through scientific collaboration and communication’ (15). While the study focused primarily on a scientific network in Southeast Asia, its report and recommendations are also relevant to scientists in other parts of the world.

Another level of communication that is critical for crisis prevention and response is international diplomacy. Sunway University has close links with universities across the Association of Southeast Asian Nations (ASEAN) and has served as a hub to the ASEAN Young Scientists Network and the International Network for Government Science Advice (INGSA)-Asia, both of which have been useful platforms for knowledge dissemination between countries and between scientists and policymakers within countries. Through the ASEAN Young Scientists Network, Sunway University has played a particularly critical role in supporting establishment of three important initiatives: 1) ASEAN Science Leadership Programme (ASEAN-SLP) that promotes inclusive leadership training and practice; 2) ASEAN Emerging Research Conference (ASEAN-ERC) that encourages the diversity of various domains of knowledge and research to converge through the lens of the ASEAN perspective; and 3) ASEAN Responsible Conduct of Research (ASEAN-RCR) that promotes mainstreaming of research integrity in the SE Asia research and development ecosystem. Through INGSA-Asia, various pilot initiatives have been established to build science advice capacity and promote the use of scientific evidence in informing policy at all levels of government in the region. For example, the INGSA Asia Grassroots Science Advice Promotions Awards have provided opportunities for individuals and stakeholders in the region to be involved in knowledge promotion activities across various themes ranging from open science, agrobusiness, water management, climate change and disasters, and women in science. Currently, INGSA Asia is working with the US National Academies of Science, Engineering and Medicine on a project to develop a guide on the prevention and mitigation of zoonotic spillovers in the live animal supply chain in the region (16).

Notwithstanding this progress, some important challenges that institutions such as Sunway University must confront when it comes to international collaboration are to ensure: (1) their sustainability beyond pilot projects; (2) academic outputs are accessible and useable by various consumers of knowledge such as policymakers and the general public; and (3) tangible impacts are measured beyond surrogate markers of academic achievement.

## 6. Conclusion

The end of COVID-19 may already be within sight. However, the harsh lessons that this pandemic has forced upon us cannot be simply forgotten and the gains that were made as a result must be sustained. Academic institutions such as Sunway University have a vital role to play in ushering in the recovery process and enhancing societal preparedness for future crises—through learning, research, advocacy, and innovation. Universities can start by creating an enabling environment for transdisciplinary collaboration, as public and planetary health challenges such as pandemics and the climate emergency cannot be successfully addressed through disciplinary siloes but rather through deepened collaboration between disciplines and with other economic and social sectors. Moreover, these health threats are rapidly evolving, and urgent action is needed now given the ever-increasing proximity of what we used to refer to as “tomorrow’s problems.” Therefore, universities must embrace a renewed emphasis on rapid transmission and translation of knowledge to the world of policy and practice, in order to solve problems and make real-world change.

## Author contributions

ER conceived the paper. ER and RQ created the draft. JM, OL-H, AV, and SP provided inputs and reviewed the draft. All authors contributed to the article and approved the submitted version.

## Conflict of interest

The authors declare that the research was conducted in the absence of any commercial or financial relationships that could be construed as a potential conflict of interest.

## Publisher’s note

All claims expressed in this article are solely those of the authors and do not necessarily represent those of their affiliated organizations, or those of the publisher, the editors and the reviewers. Any product that may be evaluated in this article, or claim that may be made by its manufacturer, is not guaranteed or endorsed by the publisher.

## References

[1] Wenham C Katz R Birungi C Boden L Eccleston-Turner M Gostin L. Global health security and universal health coverage: from a marriage of convenience to a strategic, effective partnership. BMJ Glob Health. (2019) 4:e001145. doi: 10.1136/bmjgh-2018-001145, PMID: 30713747 PMC6340060

[2] Carlson CJ Albery GF Merow C Trisos CH Zipfel CM Eskew EA. Climate change increases cross-species viral transmission risk. Nature. (2022) 607:555–62. doi: 10.1038/s41586-022-04788-w, PMID: 35483403

[3] Jakoet-Salie A Ramalobe K. The digitalization of learning and teaching practices in higher education institutions during the Covid-19 pandemic. Teach Publ Admin. (2022). doi: 10.1177/01447394221092275

[4] Ball P. The lightning-fast quest for COVID vaccines - and what it means for other diseases. Nature. (2021) 589:16–8. doi: 10.1038/d41586-020-03626-1, PMID: 33340018

[5] Whitmee S Haines A Beyrer C Boltz F Capon AG de Souza Dias BF. Safeguarding human health in the Anthropocene epoch: report of the Rockefeller Foundation-lancet commission on planetary health. Lancet (London, England). (2015) 386:1973–2028. doi: 10.1016/S0140-6736(15)60901-1, PMID: 26188744

[6] Mahmood J Guinto RR Lacey-Hall O Faiesall M Poppema S Lee E. Safeguarding planetary health for Southeast Asia's future children. Lancet Planetary Health. (2022) 6:e295–6. doi: 10.1016/S2542-5196(22)00068-7, PMID: 35397214 PMC8986167

[7] Planetary Health Alliance. Sao Paulo declaration on planetary health (2021). Available at: https://www.planetaryhealthalliance.org/sao-paulo-declaration (Accessed January 15, 2023).

[8] Raworth K. Doughnut Economics: Seven Ways to Think Like a 21st Century Economist, Broadway, New York City, New York USA: Random House Publishers. (2017).

[9] Planetary Health Alliance. Available at: https://www.planetaryhealthalliance.org/planetary-health (Accessed January 15, 2023).

[10] World Health Assembly. Resolution WHA69.1: strengthening essential public health functions in support of the achievement of universal health coverage. In: Sixty-ninth world health assembly, Geneva, (2016). Resolutions and decisions, annexes. Geneva: World Health Organization (2016). Available at: https://apps.who.int/gb/ebwha/pdf_files/WHA69-REC1/A69_2016_REC1-en.pdf#page=27 (Accessed January 15, 2023).

[11] 21st century health challenges: can the essential public health functions make a difference? WHO (2021). Available at: https://www.who.int/publications/i/item/9789240038929 (Accessed January 15, 2023).

[12] Unpublished data. Background research and analysis conducted as part of the preparatory phase and business plan development for the Sunway university MPH programme. (2021).

[13] McAdam M Debackere K. Beyond ‘triple helix’ toward ‘quadruple helix’ models in regional innovation systems: implications for theory and practice. R&D Manag. (2018) 48:3–6. doi: 10.1111/radm.12309

[14] The Lancet Infectious Diseases. The COVID-19 infodemic. Lancet Infect Dis. (2020) 20:875. doi: 10.1016/S1473-3099(20)30565-X, PMID: 32687807 PMC7367666

[15] Addressing inaccurate and misleading information about biological threats through scientific collaboration and communication in Southeast Asia. (2022). Available at: https://nap.nationalacademies.org/catalog/26466/addressing-inaccurate-and-misleading-information-about-biological-threats-through-scientific-collaboration-and-communication-in-southeast-asia (Accessed January 15, 2023).

[16] Countering zoonotic spillovers of high consequence pathogens (HCP). Available at: http://ingsa.org/nasem-zoonosis/ (Accessed January 15, 2023).

